# Physiological and transcriptomic response of enriched anammox culture upon elevated hydrazine exposure

**DOI:** 10.1007/s10532-025-10132-6

**Published:** 2025-05-05

**Authors:** Tugba Sari, Kozet Yapsakli, Deniz Akgul, Bulent Mertoglu

**Affiliations:** 1 Department of Bioengineering, Marmara University, Goztepe, 34722 Istanbul, Türkiye; 2 Department of Environmental Engineering, Marmara University, Goztepe, 34722 Istanbul, Türkiye

**Keywords:** Anammox, Anammox metabolism, Biological wastewater treatment, Environmental biotechnology, Hydrazine, Metatranscriptomics

## Abstract

Anammox has emerged as a cost-effective and eco-friendly biological treatment technology for high-strength wastewater, and hydrazine (N_2_H_4_) is a unique intermediate in the anammox metabolism. This study presents the first investigation into the genetic responses of anammox bacteria to elevated N_2_H_4_ concentrations, offering critical insights into their potential for sustainable environmental applications. In this scope, anammox cultures were exposed to high levels of N_2_H_4_ (up to 3 g/L) over a short-term period to evaluate their nitrogen treatment capacity and transcriptional responses. The results indicated that anammox activity continued at N_2_H_4_ concentrations of 1.88 g/L or less. However, acute N_2_H_4_ exposure significantly downregulated key genes, such as acetyl-CoA synthase beta and delta subunits, hydrazine synthase, hydrazine dehydrogenase, and hydroxylamine oxidoreductase, except for AAA family ATPase. Overall, high exogenous N_2_H_4_ concentrations severely constrained the metabolism and survival of anammox bacteria at a molecular level. Understanding the genetic responses of anammox bacteria to elevated N_2_H_4_ concentrations is crucial for optimizing their application in further anammox-based technologies. Future studies should focus on improving the resilience of anammox bacteria to high N_2_H_4_ concentrations, thereby broadening their applicability in engineered wastewater treatment and biotechnological processes while maintaining system stability and efficiency.

**Supplementary Information:**

The online version contains supplementary material available at 10.1007/s10532-025-10132-6.

## Introduction

Since World War II, hydrazine (N_2_H_4_) and its derivatives have versatile applications in various industries, including space and aerospace, military, environmental, agrochemical, and pharmaceuticals, as well as being an important raw material for many products in the industries of textiles, chemicals, polymers, and plastics (Patil and Rattan 2014). Currently, N_2_H_4_ and its derivatives are commercially produced exclusively through chemical processes (Nikhitha and Saibabu 2010). However, these processes have several disadvantages, such as low efficiency, high energy demands (Dirtu et al. 2006), and causing significant environmental pollution due to the salt by-products produced (Feng et al. 2021). Since the formed salts exist alongside other impurities in the solution, they often cannot be recycled or reused. Thus, they are mostly deposited in the environment, causing pollution risk (Li et al. 2020). For this reason, research studies on the recovery of industrial salts are also carried out (Alam et al. 2022; Feng et al. 2021; Li et al. 2020). However, due to the remarkable properties of N_2_H_4_, such as its energy capacity, finding alternative chemicals for similar purposes is difficult (Negri and Grund 2015). As a result, there is increasing interest in developing more eco-friendly and sustainable approaches to N_2_H_4_ production.

On the other hand, N_2_H_4_ removal from wastewater is of paramount importance because it is an extremely toxic and potentially carcinogenic substance (Eimoori et al. 2020; Eun et al. 2020). The National Toxicology Program has listed N_2_H_4_ as *reasonably anticipated to be human carcinogens* in its last report (NTP 2021). Since N_2_H_4_ can persist in water under certain conditions, various chemical techniques, such as catalytic degradation by employing different oxidizing agents or catalysts and chemical oxidation followed by dilutions, can be applied (Eun et al. 2020). Formerly, biological remediation of N_2_H_4_ has been suggested as a cost-effective and environmentally friendly alternative (Eimoori et al. 2020). Although research has been conducted, it has been formerly reported that it is a chemical that is too toxic for many (micro)organisms to tolerate under oxic conditions (Agteren et al. 1998). Furthermore, until the discovery of anaerobic ammonium oxidation (anammox) bacteria (Mulder et al. 1995), N_2_H_4_ degradation under anaerobic conditions was not known. It has recently been revealed that anammox bacteria show great tolerance to external N_2_H_4_ and exhibit simultaneous removal profiles for ammonia nitrogen (NH_4_^+^-N), nitrite nitrogen (NO_2_^−^-N), and N_2_H_4_ (Sari et al. 2024b).

Since its exploration, anammox bacteria have been identified as major contributors to nitrogen turnover in the global nitrogen cycle (Peeters and van Niftrik 2019). The anammox process is currently regarded as a sustainable and cost-saving technology due to its reduced greenhouse gas emissions, the absence of a need for an external carbon supply, lower oxygen consumption, and minimal sludge production during operation (Wu et al. 2022). In the anammox metabolism (Kartal and Keltjens 2016), nitrite reductase (Nir) first catalyzes the nitrite to nitric oxide (NO) (Eq. 1). NO further reacts with ammonium to form N_2_H_4_ through a unique hydrazine synthase (HZS) (Eq. 2), a unique characteristic of anammox bacteria. In the final step, N_2_H_4_ is oxidized to dinitrogen gas by the enzyme hydrazine dehydrogenase (HDH) (Eq. 3).

1 $$ {\text{NO}}_{{2}}^{ - } + {\text{ 2H}}^{ + } + {\text{ e}}^{ - } \to {\text{NO }} + {\text{ H}}_{{2}} {\text{O }}\left( {E_{0} ^{\prime} = \, + 0.{\text{38 V}}} \right) $$

2 $$ {\text{NO }} + {\text{ NH}}_{{4}}^{ + } + {\text{ 2H}}^{ + } + {\text{ 3e}}^{ - } \to {\text{N}}_{{2}} {\text{H}}_{{4}} + {\text{ H}}_{{2}} {\text{O }}\left( {E_{0} ^{\prime} = \, + 0.0{\text{6 V}}} \right) $$

3 $$ {\text{N}}_{{2}} {\text{H}}_{{4}} \to {\text{ N}}_{{2}} + {\text{ 4H}}^{ + } + {\text{ 4e}}^{ - } \left( {E_{0} ^{\prime} = \, - 0.{\text{75 V}}} \right) $$

As N_2_H_4_ is a notable intermediate in metabolism (Kartal and Keltjens 2016), many studies so far have focused on the trace N_2_H_4_ addition into anammox systems for various purposes. The externally added N_2_H_4_ into the anammox reactors was reported to shorten the start-up period of the process (Ganesan and Vadivelu 2019; Miodonski et al. 2019) and improve the nitrogen removal capacity of the anammox bacteria (Ma et al. 2018a; Zekker et al. 2021). Moreover, following (partial) inhibition of enriched anammox culture caused by several factors such as salinity, Cr(VI) and nitrite, the supplementation of N_2_H_4_ aids in the recovery of anammox activity (Bettazzi et al. 2010; Li et al. 2023; Ma et al. 2018a; Qu et al. 2023; Zekker et al. 2012). In anammox metabolism, N_2_H_4_ consumption and ATP production are intertwined reactions. The electrons released due to the N_2_H_4_ oxidation are used in ATP production. The produced ATP is further used in cellular activities such as cell division and maintenance of cell integrity. Therefore, N_2_H_4_ can be considered an energy source for anammox metabolism (Ganesan and Vadivelu 2020). Furthermore, from a different perspective, some studies have highlighted N_2_H_4_ biosynthesis in anammox metabolism by chemically or physically manipulating the anammox systems (Oshiki et al. 2020; Qiao et al. 2016; Sari et al. 2024a). However, long-term N_2_H_4_ production was not observed under conditions where a maximum of 55 mg/L N_2_H_4_ was obtained in the anammox system. (Sari et al. 2024a). On the other hand, literature reports a maximum supplemented N_2_H_4_ dosage of 98.7 mg/L that does not inhibit anammox activity and even enhances nitrogen removal performance (Yao et al. 2016). But still, biological N_2_H_4_ production is in its early stages but holds promising potential as an ecological alternative. Furthermore, the resilience of anammox bacteria to external N_2_H_4_ and/or N_2_H_4_ accumulation in the anammox metabolism might be improved by genetic engineering from an intriguing perspective. At present, even though engineered microbial strains are commonly utilized in various fields, such as the development of sustainable biofuels (Arora and Fatima 2024), to the best of our knowledge, no studies have yet investigated the potential of genetically modifying anammox metabolism. Up until now, the primary focus of anammox bacteria has been their utilization for pollutant ammonia elimination from wastewater (Ponce-Jahen et al. 2024). Beyond this conventional use, anammox applications might be broadened considering their N_2_H_4_ degrading ability (Sari et al. 2024b) and potential hydrazine bioproduction as a bioenergy source (Oshiki et al. 2020). Expanding research and applications involving these microorganisms could strengthen global sustainability efforts in environmental management, the mitigation of N_2_H_4_ pollution, and innovative wastewater treatment solutions.

Although N_2_H_4_ might adversely affect the anammox process at elevated concentrations (Yuan et al. 2024), the metabolic and transcriptomic responses of anammox bacteria to N_2_H_4_ exposure remain unclear. Addressing this knowledge gap is critical for understanding fundamental anammox research and developing practical solutions, particularly in light of potential long-term applications in N_2_H_4_-rich environments. In this context, the overarching aim of this study was to seek anammox response to high concentrations of N_2_H_4_ and to comprehensively understand the N_2_H_4_ effect on anammox metabolism. From this point of view, for the first time in literature, anammox bacteria were exposed to N_2_H_4_ up to 3 g/L in a short-term period, and their nitrogen removal capacities (ammonia nitrogen, nitrite nitrogen, hydrazine) were assessed. The response of the enriched anammox culture at elevated N_2_H_4_ concentrations was also investigated at gene expression levels using metatranscriptomic techniques. The results provided key insights into the functional profile of the anammox community, revealing significant transcriptional shifts, particularly in genes associated with central anammox metabolism and energy production. These findings contribute to a deeper understanding of anammox metabolism, offering crucial implications for the development and optimization of anammox-based biotechnologies.

## Material and methods

### Enrichment of anammox culture

The experimental setup of the enrichment anammox bioreactor formerly described was used with minor modifications (Sari et al. 2022). A lab-scale, 2 L side-arm bioreactor was established for anammox culture enrichment. Seed sludge was obtained from an ongoing continuous up-flow anammox reactor that has been operating continuously for over 13 years. A mixture of N_2_ and CO_2_ gases in a ratio of 95%:5% was introduced to the bioreactor to create an anoxic environment, which is essential for the growth of anammox bacteria. NaHCO_3_ (1.04 g L^−1^) was added to the synthetic wastewater to buffer the pH. Anammox bacteria use inorganic carbon provided by both CO_2_ and NaHCO_3_. Synthetic wastewater was prepared daily, as previously documented (Sari et al. 2022). Before feeding, the influent was purged with N_2_ gas to remove dissolved oxygen and ensure anoxic conditions within the reactor. Stock solutions of (NH_4_)_2_SO_4_ and NaNO_2_ were utilized to provide NH_4_^+^-N and NO_2_^−^-N in the synthetic wastewater feed at a ratio of 1:1.15. Following the initial reactor operation, NH_4_^+^-N and NO_2_^−^-N concentrations were adjusted to 50 mg/L and 55 mg/L, respectively. As the anammox bacteria adapted to their new environment, influent NH_4_^+^-N and NO_2_^−^-N concentrations were increased to 100 mg/L and 115 mg/L, respectively, in the enrichment period. The anammox bioreactor was operated in sequencing batch reactor (SBR) mode for a 24 h cycle with a 20 min fill time, 22.7 h reaction time, 40 min settling time, and 20 min effluent withdrawal time. During the operation, the temperature was maintained at 36 ± 0.5 °C, while the hydraulic retention time was set at 2 days. At the end of the enrichment period, only *Candidatus* Kuenenia stuttgartiensis was identified in the anammox culture, accounting for 82.9 ± 0.7% of the microbial population.

### Short-term experiments

To examine the response of the enriched anammox culture in a short-term manner, batch tests were performed. Amber serum flasks with 52 mL working volume were used during the study. Volatile suspended solids (VSS) were adjusted to be 1.90 ± 0.1 g/L based bacterial treatment capacity that could achieve ≥ 75% removal for both NH_4_^+^-N and NO_2_^−^-N in 12 h. Biomass was directly taken from the enrichment bioreactor by syringe and transferred to the serum flasks. The composition of synthetic wastewater was the same as that of the enrichment period. Anammox bacteria were subjected to five different N_2_H_4_ dosages (Table 1) in the form of N_2_H_6_SO_4_ (Merck, USA).

**Table 1:** Operational conditions in short-term experiments

Experiment name^a^	Operation time (h)	Applied N_2_H_4_ dosage^b^	pH range^c^
S1	24	99 ± 0.5 mg/L	7.65–8.59
S2	24	439 ± 0.4 mg/L	7.66–8.50
S3	24	1.05 ± 0.003 g/L	7.56–8.41
S4	24	1.88 ± 0.01 g/L	7.69–7.96
S5	24	3.06 ± 0.02 g/L	7.42–7.60

^a^Each condition was conducted in triplicate; ^b^Data represents average ± standard error; ^c^Data represents pH variations in the batch incubation

It should be noted that N_2_H_4_ is a rapidly degraded chemical under aerobic conditions (Rattan and Patil 2014). Hence, every stage involved in preparing the serum flasks was carried out in an anoxic environment. Moreover, N_2_H_6_SO_4_ addition significantly lowers the pH of the solutions since it reacts with O_2_ to form sulfuric acid (Singaravelan and Alwar 2014). Thus, the feed was always purged by N_2_ gas at first to remove dissolved oxygen. N_2_H_4_ was also provided into serum flasks from either freshly prepared N_2_H_4_ stock solution by oxygen-free deionized water or in powder form. After the preparation of the flasks, the contents were again flushed with N_2_ for 3 min. Finally, the batch flasks were sealed with a rubber stopper and aluminum crimp and placed in incubator at 36 ± 0.5 °C for 24 h incubation period. The samples were taken every 2 h to carry out analytical analyses, including NH_4_^+^-N, NO_2_^−^-N and N_2_H_4_. The initial pH of each serum bottle was also measured at the first sampling (Table 1); therefore, 1.33 ± 0.01 mL sample was collected from batch bottles at t = 0 h. Other samples were withdrawn from the systems to be less than 1 mL. Additionally, the negative control was established in the absence of enriched anammox culture to address the stability of chemicals (NH_4_^+^-N, NO_2_^−^-N and N_2_H_4_) in the synthetic wastewater over time. Each condition was performed in triplicate.

### RNA isolation and sequencing

Based on the results of the short-term experiments, additional batch bottles were set up to simulate control and 2 g/L experiments for metatranscriptomic analysis using the same experimental procedures. These experiments were performed in duplicate. Mixed liquor sludge samples (MLVSS) were collected from the control bottles when NH_4_^+^-N and NO_2_^−^-N removal efficiencies were 35.8 ± 4.8% and 36.2 ± 4.4%, respectively. Samples were taken from the bottles exposed to 2 g/L N_2_H_4_, when at least 25% NO_2_^−^-N treatment efficiency was achieved. RNA from the samples was isolated using the Quick-RNA Miniprep Kit (Zymo Research, USA) according to the manufacturer’s instructions. Lifescience Research and Application Center (Ankara, Türkiye) performed pair-end sequencing of the samples using Illumina Stranded Total RNA Prep, Ligation with Ribo-Zero Plus Kit according to the Reference Guide, and the NovaSeq 6000 system (Illumina, USA).

### Bioinformatics processing

Metatranscriptomic analysis was conducted by the Lifescience Research and Application Center (Ankara, Türkiye). Following quality filtering, high-quality sequencing reads in “.fastq” format were taxonomically classified using Kraken2 v2.1.3 software to generate operational taxonomic units (OTUs). A Sankey diagram, a visualization technique, was used to illustrate species distribution patterns. Reads assigned to the *Candidatus* Kuenenia stuttgartiensis species were subsequently identified. After quality filtering and taxonomic classification, sequencing reads in “.fastq” format were subsequently aligned to the *Candidatus* Kuenenia stuttgartiensis reference genome (ASM1106654v1, GCF_011066545.1) using Bowtie2 v2.5.3 software. The obtained alignments were then converted from SAM to the more efficient BAM format and sorted using SAMtools v1.19.0. The generation of alignment statistics is given in Table S1 (in Supplementary Information (SI) 1). RNA-seq data, stored in the BAM file format, were analyzed to quantify gene expression in *Candidatus* Kuenenia stuttgartiensis. The featureCounts v2.0.7 software was employed to generate read counts by aligning the sequencing reads to its annotated genome. This process facilitates the identification of genes that are actively transcribed under the experimental conditions used in the RNA-seq experiment. This data serves as the foundation for further analysis of differential gene expressions. To identify differentially expressed genes (DEGs) between experimental groups, DeSeq2 v1.42.0 software was utilized. A cutoff of |log_2_(FoldChange)|≥ 1.0 and adjusted *p*-value (*p*-adj) ≤ 0.01 were applied to define DEGs. DeSeq2 analysis revealed statistically significant differential expressions between the experimental and control groups. The heat map and volcano plot analysis were used for the visualization of the DEGs. In addition, gene ontology (GO) enrichment analysis was conducted to provide a set of annotations to comprehensively describe the properties of genes and gene products. Table S2 (in SI 1) provides a summary of all software tools and databases used in the bioinformatics analyses. The raw reads were also deposited in the NCBI Sequence Read Archive (SRA) database (Accession Numbers: SAMN47400858, SAMN47400859, SAMN47400860, and SAMN47400861).

### Analytical analyses

VSS, NH_4_^+^-N, and NO_2_^−^-N were analyzed following the Standard Methods (APHA 1998). Samples were pretreated according to the Crosby method to prevent interference from N_2_H_4_ in NH_4_^+^-N measurements (Crosby 1968). N_2_H_4_ concentrations were determined using the p-dimethylaminobenzaldehyde method, adapted from the ASTM Manual of Industrial Water, with HydraVer 2 reagent (HACH, USA). Due to the rapid degradation of N_2_H_4_ under oxic conditions, analysis was performed immediately after sampling (Rattan and Patil 2014). In addition, samples were pretreated with 0.5% sulfamic acid before N_2_H_4_ analysis to mitigate NO_2_^−^-N interference (George et al. 2008). The pH and dissolved oxygen levels in the samples were monitored using a digital portable multimeter kit (HACH, USA).

### Statistical analyses

Results were expressed as the mean ± standard error. The Shapiro–Wilk test was applied to examine the normality of quantitative variables using GraphPad Prism (version 7.00). A one-factorial analysis of variance (one-way ANOVA) was employed to compare the means between all treatment groups. In order to reveal an individual difference between the two treatment groups, a paired t-test was typically conducted. For all analysis, Microsoft Excel was used.

## Results and discussion

### Short-term response to hydrazine

In the literature, the maximum supplemented N_2_H_4_ dosage to the anammox system was 98.7 mg/L, which did not inhibit the anammox activity in a brief period and even accelerated the total nitrogen removal rate of the anammox bacteria (Yao et al. 2016). Hence, in the current study, the applied N_2_H_4_ dosages were chosen to be ≥ 99 mg/L during the short-term experiments (Table 1). Negative control studies revealed that, in the absence of enriched anammox culture, no tested compounds were consumed (Fig. S1 in SI 1). In the batch tests, the N_2_H_4_ consumption by the enriched anammox culture increased over the incubation time as the applied N_2_H_4_ dosages were increased, except for S5 (Fig. S2a in SI 1). In this experimental group (S5), no N_2_H_4_ consumption was observed. In this respect, exposure of anammox bacteria to a substantially high substrate concentration might have affected the metabolic pathways related to N_2_H_4_ consumption (“Effects of exogenous N2H4 on anammox metabolism” section).

**Fig. 1:**
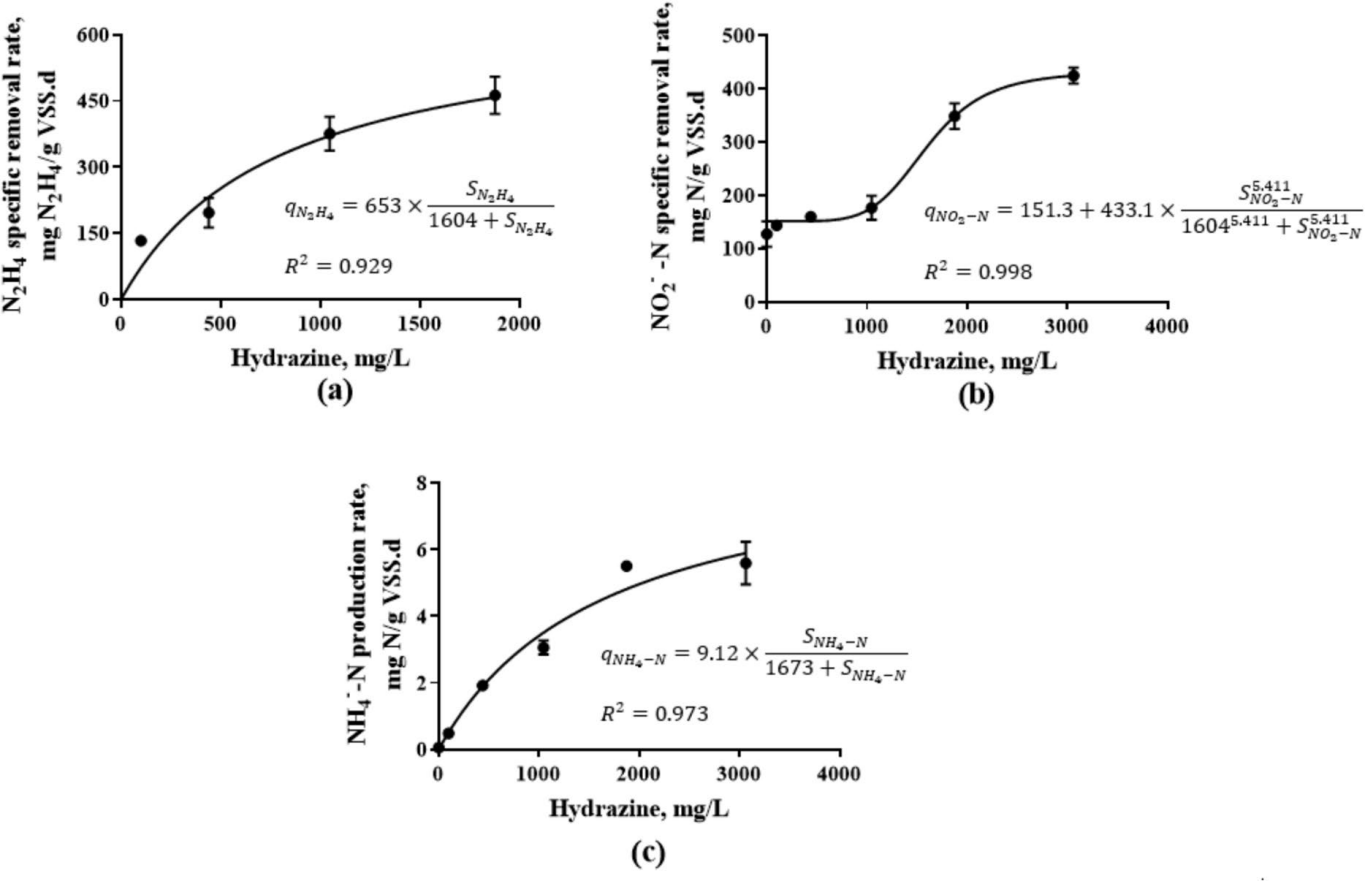
Short-term effect of exogenous N_2_H_4_ on anammox process. **a** N_2_H_4_ consumption rate, **b** NO_2_^−^-N consumption rate, **c** NH_4_^+^-N consumption rate

Batch short-term assays provided a visual representation of exogenous N_2_H_4_ consumption over 24 h. However, the rate of N_2_H_4_ consumption was determined based on the fast initial rate. As shown in Fig. 1a, the N_2_H_4_ removal rate increased remarkably with increasing applied N_2_H_4_ dosages, eventually leveling off at a concentration of 1.88 ± 0.01 g/L. The difference between means of treatment groups was also found to be statistically significant as determined by one-way ANOVA [*F*_3,8_(0.05) = 14.2, *p* = 0.001]. However, when compared to S1, there was no significant increase in the N_2_H_4_ consumption rate in S2 (α = 0.05, *p* = 0.159 > 0.05 for μ1 = μ2). Along with the applications of 1.05 ± 0.003 g/L N_2_H_4_ and 1.88 ± 0.01 g/L N_2_H_4_, a significant boost was observed in the N_2_H_4_ consumption rate (α = 0.05, *p* = 0.0176 < 0.05 for μ1 = μ2 for S3; α = 0.05, *p* = 0.0102 < 0.05 for μ1 = μ2 for S4). The overall findings have pointed out that Monod-like kinetics was observed for N_2_H_4_ consumption at applied concentrations ranging from 99 ± 0.5 mg/L to 1.88 ± 0.01 g/L (Fig. 1a). Kinetic parameters for N_2_H_4_ consumption were determined in the presence of 99.2 ± 5.7 mg/L NH₄^+^-N and 112 ± 0.7 mg/L NO₂^−^-N. The maximum specific N_2_H_4_ consumption rate (k_N₂H₄_) was predicted to be 653 ± 166 mg/g VSS.d, with a half-saturation constant (K_N₂H₄_) of 796 ± 484 mg/L. In comparison to the kinetic coefficients reported by Soler-Jofra et al. (2020) and Ma et al. (2018a), which were approximately one-third and one-fourth, respectively, of our determined value, the maximum rate constant in this study was significantly higher. This difference could be attributed to at least 20-fold lower N_2_H_4_ concentrations employed in the referenced studies. A high half-saturation constant found in this study (Ks = 796 mg/L) indicates a low affinity of the anammox bacteria for the substrate (N_2_H_4_). This result also implies that anammox bacteria have a high tolerance to elevated N_2_H_4_ concentrations without impairing bacterial activity. Yao et al. (2015) obtained a Ks constant of 10.4 mg N/L (11.9 mg N_2_H_4_/L), while it was 0.571 mmol/L (18.3 mg N_2_H_4_/L) in the studies of Ma et al. (2018a). Besides, in this study, further increasing the N_2_H_4_ concentration to 3.06 ± 0.02 g/L resulted in a halt in N_2_H_4_ removal, indicating a possible inhibition in anammox mechanism. No known kinetic inhibition models (competitive, noncompetitive, uncompetitive, or substrate inhibition) can explain the abrupt cessation of N_2_H_4_ removal. The potential molecular-level impacts of exogenous N_2_H_4_ will be detailed in “Effects of exogenous N2H4 on anammox metabolism” section.

As for the NO_2_^−^-N removal profile of the batch systems, influent nitrite was gradually treated by enriched anammox culture in all experiment groups as in the control group. Although NO_2_^−^-N removal efficiency was equal to 33.9 ± 4.10% within 6 h in the control group, it increased to 67.4 ± 3.4% within the same period in all treatment groups, including from S1 to S5 (Fig. S2b in SI 1). The former studies have also reported that the exogenous N_2_H_4_ addition increased the nitrite degradation rate. Briefly stated, when the nitrite concentration was increased up to 3.98 mM (55.8 mg N/L) in the presence of N_2_H_4_ (1 mM, 32 mg/L), the nitrite degradation rate peaked at 12.8 mM/g VSS.d based on the curve fitting analysis (Ma et al. 2018a). In the present study, even in the absence of exogenous N_2_H_4_, a significant NO_2_^−^-N utilization rate of 127 ± 6.10 mg N/g VSS.d was observed, primarily attributed to anammox activity. At each dosage of N_2_H_4_ (≤ 1 g/L), the rate increased only progressively with each successive increment in the N_2_H_4_ concentration, exhibiting a sigmoidal shape (Fig. 1b). Further increase in N_2_H_4_ concentration created large increases in NO_2_^−^-N utilization rate. Nevertheless, at saturating substrate levels, the reaction velocity plateaus, becoming increasingly insensitive to further substrate additions. This type of sigmoidal relationship exhibiting saturation kinetics can be described by the Hill equation. Therefore, the following variable slope-sigmoidal dose–response model was selected for fitting N_2_H_4_ dependent NO_2_-^−^N utilization kinetics.

4 $${q}_{NO2-N}={q}_{0}+{k}_{NO2}\times \frac{{S}^{h}}{{EC50}_{NO2}^{h}+{S}^{h}}$$

where q_NO2-N_ is the NO_2_^−^-N utilization rate (mg N/g VSS.d), q_0_ is baseline NO_2_^−^-N utilization rate in the absence of exogenous N_2_H_4_ (mg N/g VSS.d), k_NO2_ is constant indicating the maximum utilization rate of NO_2_^−^-N (mg N/g VSS.d), EC50_NO2_ is the concentration of N_2_H_4_ that gives a response half way between baseline rate and maximum rate (mg N/L), S is N_2_H_4_ concentration (mg N/L), h is the Hill’s coefficient (unitless).

The Hill coefficient (h) is an empirical parameter reflecting the number, type, and strength of interactions among multiple substrate-binding sites on the enzyme. The Hill equation can be used to account for cooperativity of substrate binding to an enzyme (allosteric saturation curve) (Pabis et al. 2018). The allosteric saturation curve is sigmoid, not hyperbolic like a typical Michaelis Menten enzyme. Fitting experimental data into Hill’s equation resulted in a maximum specific nitrite consumption rate (k_NO2_) of 433 ± 1.12 mg/g VSS.d, an EC50_NO2_ constant of 1.60 ± 0.08 g/L. A Hill coefficient of 5.41 ± 1.12 indicates a positive level of cooperativity (h > 1) (Fig. 1b). A system’s ability to exhibit cooperativity can help to ensure consistent performance of the system even when exposed to unfavorable conditions such as low temperature, drastic changes in substrate availability or inhibitors (Pabis et al. 2018). Positive level of cooperativity in this case resulted in improved nitrite removal efficiencies despite high N_2_H_4_ levels.

The profile of NH_4_^+^-N concentration during the batch tests showed different patterns, unlike nitrite (Fig. S2c in SI 1). In the control group, NH_4_^+^-N was removed simultaneously with nitrite from the anammox system, and both substrates were completely utilized throughout the incubation period. As for the experimental group S1, only 75.6 ± 2.4% of NH_4_^+^-N was removed in 24 h. However, NH_4_^+^-N consumption decreased with increasing N_2_H_4_ dosage, and even NH_4_^+^-N accumulation was observed in the anammox systems. In the treatment group S2, a total of 18.4 ± 4.16 mg/L NH_4_^+^-N production was detected in the serum bottles during the incubation. When the applied N_2_H_4_ dosage was further increased, as in groups S3, S4, and S5, NH_4_^+^-N started to accumulate gradually in the batch bottles. NH_4_^+^-N production rate in response to increasing N_2_H_4_ concentration is shown in Fig. 1c. It has been previously documented that anammox metabolism generates ammonium from N_2_H_4_ (Carvajal-Arroyo et al. 2013). Theoretically, the N_2_H_4_ disproportionation reaction in anammox metabolism yields 1.3 mol of ammonium per mole of N_2_H_4_ (Soler-Jofra et al. 2020). In the batch tests except for S5, 1.23 ± 0.16 M NH_4_^+^ was produced per 1 M N_2_H_4_, which is quite close to the literature, considering the consumption ratio of NH_4_^+^-N:NO_2_^−^-N (1:1.146) (Lotti et al. 2014). In such a case, the anammox process should be integrated with a partial nitrification process for treating ammonia-rich wastewater containing high concentrations of N_2_H_4_, considering the major priority of the anammox process is to remove ammonia nitrogen in industry.

Additionally, if there is a low N_2_H_4_ concentration in the system, the formed NH_4_^+^ may be consumed by anammox bacteria immediately. However, at high N_2_H_4_ concentrations, NH_4_^+^ accumulation may occur due to the limit of the metabolic capacity of the bacteria. On the other hand, the mechanism of the disproportionation reaction depends on reaction rates and thermodynamic balances (Soler-Jofra et al. 2020). At low N_2_H_4_ levels, the reaction may not lead to NH_4_^+^ formation or NH_4_^+^ formation may be slower. However, at higher N_2_H_4_ concentrations, the kinetic equilibrium point of the reaction may change, which may increase NH_4_^+^ accumulation. In order to confirm these hypotheses, the reaction mechanisms and kinetic rate constants in the system may need to be examined in more detail. Besides, there are no pure cultures of anammox bacteria, all studies are carried out with enriched cultures (Peeters and van Niftrik 2019). Consequently, the complex microbial dynamics within an anammox system are one of the general limitations of anammox studies.

### Hydrazine-induced transcriptional response of anammox culture

#### Microbial diversity in biomass samples

**Fig. 2:**
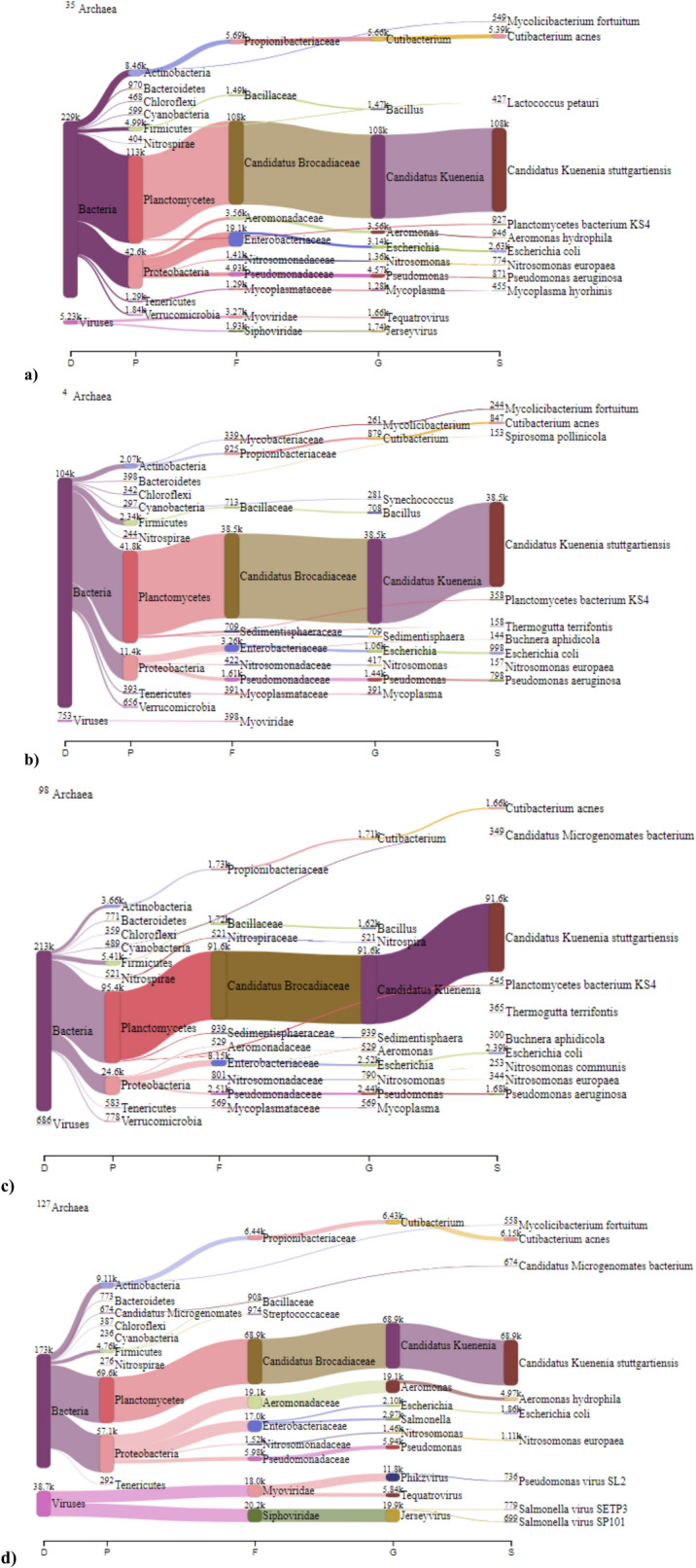
Sankey diagram depicting the distribution of identified species across. **a** control sample 1, **b** control sample 2, **c** treatment sample 1, **d** treatment sample 2. Treatment samples were exposed to 2 g/L N_2_H_4_

Since a pure culture of anammox bacteria could not be obtained, metatranscriptomic analyses were performed with enriched anammox cultures. Analysis of species diversity in the metatranscriptome confirmed transcripts from several prokaryotic organisms, including anammox bacteria. Taxonomic assignments in the biomass samples are illustrated by Sanger diagrams (Fig. 2). Besides, many RNA reads (approximately 20–50%) could not be assigned to bacteria, archaea or viruses. This may be related to the classification capabilities of the bioinformatics software used and database limitations. Additionally, unknown or undiscovered RNA read data from various microorganisms from environmental samples may have contributed to these unclassified reads. The existence of new and unknown organisms or error rates may explain this situation.

The phylum Planctomycetes (now renamed Planctomycetota) represents the majority of the microbial community in all biomass samples. Anammox bacteria, belonging to the class *Candidatus* Brocadiia (formerly Brocadiaceae), are deep branches of the Planctomycetota. All anammox bacteria have *Candidatus* (*Ca.*) status, as there are no pure cultures of them (Peeters and van Niftrik 2019). *Ca.* Kuenenia stuttgartiensis was the only identified anammox species during the experiments. In the control samples, *Ca.* Kuenenia stuttgartiensis was present at 82.9 ± 0.7% (Fig. 2a and b). Exposure to 2 g/L N_2_H_4_ caused a slight decrease in its relative abundance, reaching 77 ± 5.9% (Fig. 2c and d). Although, in the current study, the relative abundance of anammox bacteria was found to reduce as a result of short-term N_2_H_4_ exposure, they typically have long doubling times, ranging from several days to weeks (Peeters and van Niftrik 2019). Therefore, 24 h exposure to N_2_H_4_ is unlikely to alter the structure of the microbial population. To observe this, applying longer exposure periods would be more realistic (Sari et al. 2024b).

The second most abundant phylum was found to be Pseudomonadota (formerly Proteobacteria). Other phyla in the microbial community were Actinomycetota (formerly Actinobacteria), Bacteroidota (formerly Bacteroidetes), Chloroflexota (formerly Chloroflexi), Cyanobacteria, Bacillota (formerly Firmicutes), Nitrospirota (or Nitrospirae), Mycoplasmatota (formerly Tenericutes), and Verrucomicrobiota (formerly Verrumicrobia), with relatively lower abundances. Although the core anammox community in the microbial population showed differences, the coexistence of Planctomycetota, representing anammox bacteria, with Pseudomonadota, Chloroflexota, Bacteroidota, and Bacillota was previously reported (Aktan et al. 2021; Ma et al. 2018b; Zhang et al. 2019). The symbiotic relationship in the anammox consortia under various environmental conditions has also been well documented in several studies (Lawson et al. 2017; Speth et al. 2016; Ya et al. 2022). Microorganisms coexisting with anammox bacteria in the biological system engage in complementary metabolic activities. Denitrifiers, which are mostly affiliated with Proteobacteria, may use nitrate produced by anammox as an electron acceptor, while fermentative bacteria, such as Chloroflexi, may utilize decayed microorganisms, including anammox, to provide a carbon source for heterotrophs (Pereira et al. 2017). Besides, the Chloroflexi play a key role in sludge granulation, flocculation, and biofilm formation due to their filamentous growth (Bovio-Winkler et al. 2023). Similarly, Bacteroidota contributes by scavenging EPS and degrading cell debris in the system (Oshiki et al. 2022). Firmicutes aid in N_2_O consumption and possess genes for denitrification and ammonification, enabling them to thrive in anaerobic, nutrient-rich conditions and support nitrogen removal in bioreactors (Pekyavas and Yangin-Gomec 2019).

#### Overview of differential gene expression analysis

**Fig. 3:**
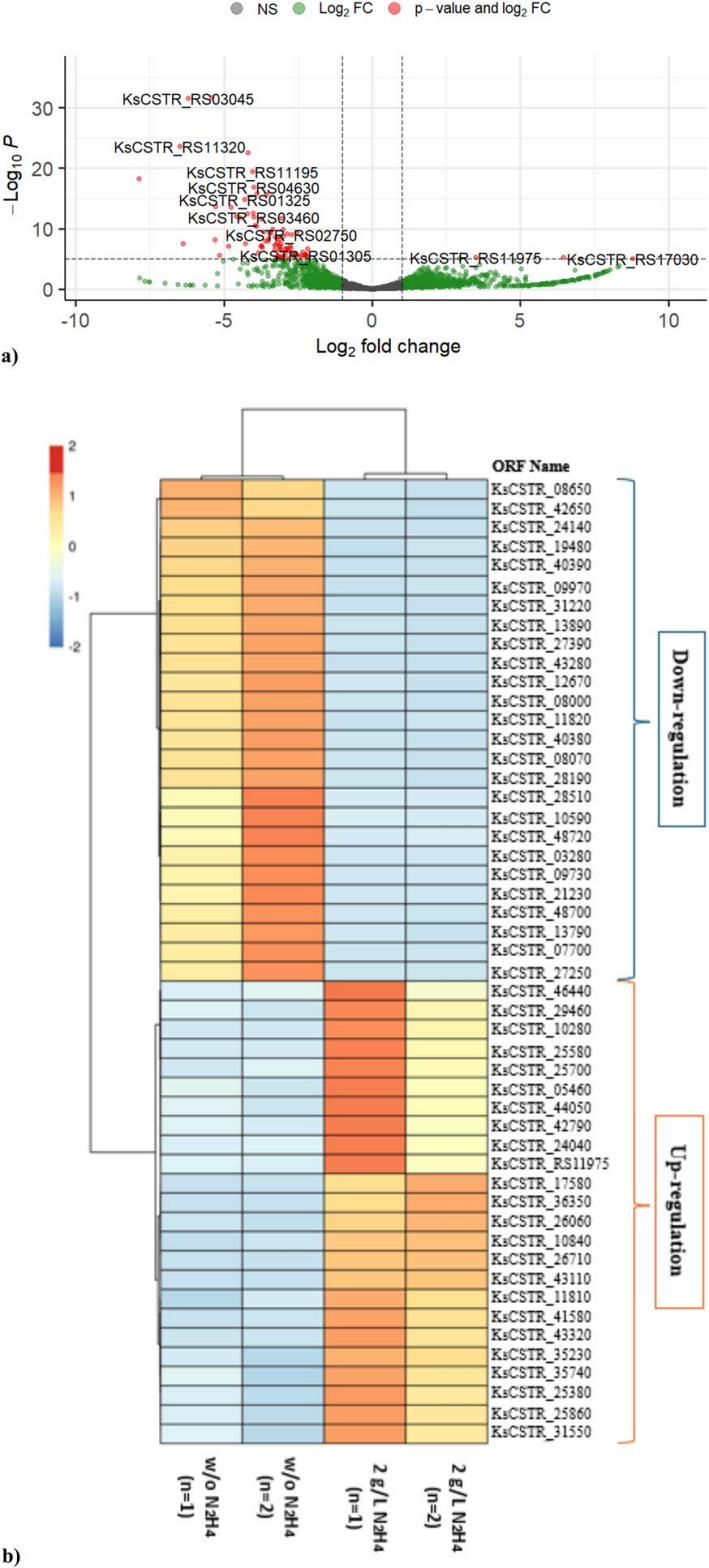
Differentially expressed genes across control and treatment conditions. **a** Volcano plot representing DEGs; **b** Heatmap illustrating the expression profiles of the top 50 up-regulated and down-regulated. DEGs are represented as points, with those exhibiting significant upregulation on the right side, those with significant downregulation on the left side, and non-significantly (NS) expressed genes clustered around the center. For the locus tag, see SI 2

Differential gene expression analysis has become a widely used approach to improve our understanding of the quantitative variations in gene expression levels. A volcano plot analysis was used to visualize the DEGs (Fig. 3a). This plot shows the relationship between the statistical significance (often represented as the -log_10_ of the *p*-value) on the y-axis and the magnitude of change (usually the log_2_(FoldChange) on the x-axis (Fig. 3a). Therefore, this analysis helps to identify genes that are both statistically significant and biologically relevant, providing valuable insight into gene expression changes across experimental conditions. From the overall perspective, in the volcano plot, a total of 3062 variables were determined. A total of 54 genes were upregulated, while 199 genes were downregulated in response to exogenous N_2_H_4_ addition. The expression profiles of the 25 most DEGs, including both up- and down-regulated transcripts, were presented by a heatmap illustration (Fig. 3b). A complete list of these genes and their expression levels can be found in SI 2. Besides, a lollipop plot was used to illustrate the GO functional enrichment (Fig. S3 in SI1). This analysis is also briefly explained in Text S1 in SI1.

#### *Effects of exogenous N*_*2*_*H*_*4*_* on anammox metabolism*

The results have shown that high level of N_2_H_4_ (~ 2 g/L) stress significantly decreased the expression dynamics in the enriched anammox culture (Table 2). It indicates that anammox metabolism, including HAO-like proteins, N_2_H_4_ synthesis, ATP synthesis and energy conservation, acetyl-CoA pathway, and substrate trafficking, are vulnerable to shock loads of N_2_H_4_ at high concentrations.

**Table 2:** Overview of the most important anammox genes detected in the transcriptomes

Annotated function	ORF name	*gene*	Gene description	Gene ontology
HAO-like proteins	KsCSTR_43280	*hao*	hydroxylamine oxidase	**CC-** [GO:0044222]; **MF-** [GO:0020037]; [GO:0033740]; [GO:0042802]; [GO:0046872]; **BP-** [GO:0019331]; [GO:0006809]; [GO:0070207]
	KsCSTR_40830	*hao*	multiheme c-type cytochrome	**MF-** [GO:0033740]
	KsCSTR_46980	*hdh*	hydrazine dehydrogenase	**CC-** [GO:0016020]; **MF-** [GO:0033740]
	KsCSTR_11820	*hao*	hydrazine dehydrogenase	**MF-** [GO:0033740]
	KsCSTR_49500	–	cytochrome c	**CC-** [GO:0044222]; [GO:0070469]; **MF-** [GO:0009055]; [GO:0020037]; [GO:0046872]
	KsCSTR_29630	*hao*	multiheme c-type cytochrome	**CC-** [GO:0016020]
N_2_H_4_ synthesis	KsCSTR_12670/ KsCSTR_28190	*hzsB*	HZS subunit beta	–
	KsCSTR_12680/ KsCSTR_28200	*hzsC*	HZS subunit gamma	**MF-** [GO:0009055]; [GO:0020037]; [GO:0046872]
	KsCSTR_12690/ KsCSTR_28210	*hzsA*	HZS subunit alpha	**MF-** [GO:0016491]
Energy metabolism	KsCSTR_49510	–	hypothetical protein^1^	–
	KsCSTR_29670	–	FAD-dependent oxidoreductase	**MF-** [GO:0051536]; [GO:0046872]; [GO:0016491]
	KsCSTR_29640	–	multiheme c-type cytochrome	**CC-** [GO:0016020]
	KsCSTR_28140	–	multiheme c-type cytochrome	**MF-** [GO:0009055]; [GO:0020037]; [GO:0046872]
	KsCSTR_28150	–	Hypothetical (Hepta heme) protein	**CC-** [GO:0016020]
	KsCSTR_28170	*atoC*	sigma-54 dependent transcriptional regulator	**MF-** [GO:0005524]; [GO:0016887]; [GO:0043565]; **BP-** [GO:0000160]; [GO:0006355]
	KsCSTR_28180	–	hypothetical protein^2^	–
	KsCSTR_40380	*naxS*	cytochrome c	**MF-** [GO:0009055]; [GO:0020037]; [GO:0046872]
	KsCSTR_40390	*naxL*	cytochrome c	**MF-** [GO:0009055]; [GO:0020037]; [GO:0005506]
	KsCSTR_19180	–	hypothetical protein^3^	
	KsCSTR_10280	–	c-type cytochrome	**CC-** [GO:0016020]; **MF-** [GO:0009055]; [GO:0020037]; [GO:0046872]
	KsCSTR_22800	–	ATP synthase subunit I	**CC-** [GO:0005886]
	KsCSTR_22820	*atpE*	ATP synthase F0 subunit C	**CC-** [GO:0005886]; [GO:0045263]; **MF-** [GO:0008289]; [GO:0046933]
	KsCSTR_22840	*atpF*	F0F1 ATP synthase subunit B	**CC-** [GO:0005886]; [GO:0045263]; **MF-** [GO:0046933]
	KsCSTR_19160	–	MoxR family ATPase	**MF-** [GO:0005524]; [GO:0016887]
	KsCSTR_20440	–	GldG family protein	**CC-** [GO:0016020]
	KsCSTR_20420	–	ATP-binding cassette domain-containing protein	**MF-** [GO:0005524]; [GO:0016887]
	KsCSTR_25000	–	AAA family ATPase	**CC-** [GO:0005737]; **MF-** [GO:0005524]; [GO:0016887]; **BP-** [GO:0034605]
	KsCSTR_21550	*exeA*	AAA family ATPase	**MF-** [GO:0016887]
	KsCSTR_25860	*exeA*	AAA family ATPase	**MF-** [GO:0016887]
	KsCSTR_31550	*exeA*	AAA family ATPase	**MF-** [GO:0016887]
	KsCSTR_35740	*exeA*	AAA family ATPase	**MF-** [GO:0016887]
Stress response	KsCSTR_48320	*hsp*	Hsp20/alpha crystallin family protein	**MF-** [GO:0051082]; **BP-** [GO:0051259]; [GO:0006457]; [GO:0009408]; [GO:0042542]; [GO:0009651]
Acetyl-CoA metabolism	KsCSTR_09660	*acsB*	acetyl-CoA decarbonylase/synthase complex subunit alpha/beta	**MF-** [GO:0018492]; [GO:0043884]; [GO:0051536]; [GO:0046872]; **BP-** [GO:0006084]
	KsCSTR_09730	*acsD*	acetyl-CoA decarbonylase/synthase complex subunit delta	**MF-** [GO:0016491]
Nitrate reductase	KsCSTR_08000	*narG*	molybdopterin-dependent oxidoreductase	**MF-** [GO:0051536]; [GO:0046872]; [GO:0008940]
Nitrite transporter	KsCSTR_14610	*focA*	formate/nitrite transporter family protein	**CC-** [GO:0016020]; **MF-** [GO:0022857]
Nitrite reductase	KsCSTR_41990	*nirD/nirG*	AsnC family transcriptional regulator	**MF-** [GO:0043565]; [GO:0016829]

Highlighted genes were upregulated, others were downregulated. Gene names and gene ontology were obtained from UniProt database. *CC* Cellular component, *MF* Molecular function, *BP* Biological process. ^1^ submitted protein name: Type-1 blue copper-containing cupredoxin; ^2^submitted protein name: Uncharacterized protein; ^3^submitted protein name: C-type heme protein

##### HAO-like proteins and energy conservation of anammox bacteria

Aerobic ammonium-oxidizing bacteria (AOB) use hydroxylamine oxidoreductase (HAO), a multiheme protein, to break down hydroxylamine and form nitrite (Hooper et al. 1997). Hydrazine oxidation has also been reported as a side activity in vitro by AOB HAO (Hooper and Nason 1965; Kartal and Keltjens 2016). As for the current understanding of anammox metabolism, HAO is one of the key enzymes in metabolism, forming NO from NH_2_OH escaping from the HZS complex (Akram et al. 2021). Up to now, in the genome of *K. stuttgartiensis*, ten HAO-related octaheme proteins encoded by kustc0458, kuste4574, kuste2479, kusta0043, kustc1061, kustc0694, kustd1340, kustd2021, kuste2435, and kuste2457 have been identified (de Almeida et al. 2011) with different expression levels (Kartal et al. 2013). Among them, kustc1061 (KsCSTR_43280) is the first representative of HDH in *K. stuttgartiensis*, denoting hydroxylamine oxidase. It is also the most abundant HAO-like protein in the *K. stuttgartiensis* proteome and is capable of hydrazine oxidation (Eq. 3), albeit slowly and with low affinity (Kartal et al. 2011). The main activity of this HAO-like protein has been proposed to oxidize NH_2_OH to NO, not to NO_2_^−^-. However, its role is still enigmatic (Kartal et al. 2013). In the current study, KsCSTR_43280 and KsCSTR_40830 (kusta0043) were found to be downregulated (*p*-adj < 0.05) (Table 2).

Additionally, in *K. stuttgartiensis*, the genuine HDH, responsible for the last reaction of central metabolism (Eq. 3), is belonging to the HAO-like octaheme proteins (Shimamura et al. 2007), and a gene product of kustc0694 (Maalcke et al. 2016). A second gene (kustd1340, KsCSTR_11820) with a high sequence similarity to HDH (> 98% in the translated amino acid sequence) is also found in the genome of *K. stuttgartiensis*. Only *K. stuttgartiensis* cells under stress were found to express kustd1340 at the transcript level; its regulation is yet unknown (Hu et al. 2013). Probably for this reason, in the current study, KsCSTR_11820 was significantly downregulated (*p*-adj < 0.01) as a result of the high-level N_2_H_4_ exposure of anammox bacteria, in addition to the downregulation of KsCSTR_46980, another representing *hdh* gene (*p*-adj < 0.01).

The roles of kustc0458, kuste4574 (KsCSTR_29630), and kuste2479 are also interesting in metabolism (Kartal et al. 2013). Kustc0458 is linked to genes encoding a diheme cyt c (kustc0457, KsCSTR_49500) and a novel type-1 blue copper-containing cupredoxin (kustc0456, KsCSTR_49510), both of which might serve as redox partners (Kartal et al. 2013), and they were significantly downregulated in the present study (*p*-adj < 0.05) (Table 2). As for KsCSTR_29630, which is the HAO-like octaheme protein and a close homolog of kustc0458, it forms part of a Rieske-heme *b* (R/b; *bc1*) complex (de Almeida et al. 2016). As a result of short-term exposure to N_2_H_4_, both the KsCSTR_29630 gene and kuste4570 (KsCSTR_29670) and a hexaheme c-type protein (kuste4573, KsCSTR_29640) of the *bc1* complex were also downregulated (*p*-adj < 0.05) (Table 2). The *bc1* complexes also play an important role in the proposed anammox energy metabolism and are encoded in the *K. stuttgartiensis* genome by three different gene clusters, including kuste3096-3097, kustd1480-1485, and kuste4569-4574 (de Almeida et al. 2011). Although they are expressed at the transcriptional and protein levels in different amounts, kuste4569-4574 forms the major species (Kartal et al. 2013).

##### Hydrazine synthesis and ATP synthesis

In the genome of *K. stuttgartiensis*, a gene set (kuste2859-2861) has been suggested to encode for HZS (Strous et al. 2006) comprised of three subunits (alpha, beta, and gamma) (Dietl et al. 2015), which catalyzes Eq. 2. In this study, the genes encoding the proteins, including HZS subunit A (*hzsA*, KsCSTR_12690, KsCSTR_28210), HZS subunit beta (*hzsB*, KsCSTR_12670, KsCSTR_28190), and HZS subunit gamma (*hzsC*, KsCSTR_12680, KsCSTR_28200), were considerably downregulated (*p*-adj < 0.01) (Table 2 and Fig. 3b). The catalytic module of HZS is also part of a larger gene cluster (kuste2854-2861) (Kartal et al. 2011), facilitating electron donors for the HZS reaction (Kartal et al. 2013). It includes a membrane-bound, heme *b*-containing electron transfer module (kuste2855-2856) and a soluble triheme protein (kuste2854, KsCSTR_28140), potentially involved in the gathering of electrons derived from menaquinol oxidation and their delivery to HZS (Kartal and Keltjens 2016). Additionally, a sigma-54 transcriptional regulator (kuste2857, KsCSTR_28170) and an uncharacterized protein (kuste2858, KsCSTR_28180) are involved in this gene cluster. Among them, kuste2854, kuste2855 (KsCSTR_28150), KsCSTR_28170, and KsCSTR_28180 were also downregulated (*p*-adj < 0.05 for KsCSTR_28170 and *p*-adj < 0.01 for others) (Table 2).

N_2_H_4_ is a powerful reductant. Thus, in the anammox metabolism, there is a good chance that the electrons that result from N_2_H_4_ oxidation are used to encourage the generation of proton-motive force (*pmf*) (Kartal et al. 2013). Anammox bacteria possess a variety of modules to shuttle electrons to or from the quinone/quinol (Q) pool (Kartal and Keltjens 2016). NaxS (kusta0088, KsCSTR_40380) and naxL (kusta0087, KsCSTR_40390) are identified electron carriers (small soluble cyt c) (Kartal et al. 2013), and they were downregulated by high-level of N_2_H_4_ exposure (*p-adj* < 0.01) (Table 2 and Fig. 3b). Other genes encoding proteins with electron transfer activity, including KsCSTR_19180 and KsCSTR_31460, were also be found to downregulate (*p*-adj < 0.01), whereas KsCSTR_10280 was upregulated (*p*-adj < 0.05) (Table 2).

Central anammox reactions are intertwined with the energy metabolism. *Pmf* is produced by a net proton translocation via a semi-permeable membrane system as a result of the redox reactions and proton movements associated with the catabolic reactions. The membrane-bound ATP synthase complex can then use the *pmf* to promote ATP synthesis (van Niftrik 2013). In the genome of *K. stuttgartiensis*, there are four different clusters of ATPases, which are ATPase-1 (the ubiquitous H^+^-dependent F_1_F_0_-type), two closely related F-ATPases (ATPase-2 and ATPase3), and a prokaryotic V-type ATPase (ATPase-4) encoded by kuste3787-3796, kuste4592-4600, kustc0572-0579, and kuste3864-3871, respectively (de Almeida et al. 2016; van Niftrik et al. 2010).

ATPase-1 is the most abundant species in anammox bacteria and is predominantly localized at the anammoxosome and, to a lesser extent, near the outermost membrane (van Niftrik et al. 2010). In this study, only the genes involved in ATPase-1 were affected. Specifically, KsCSTR_22800, KsCSTR_22820 (kuste3790), and KsCSTR_22840 were downregulated (*p*-adj < 0.05). In addition, the genes KsCSTR_19160, KsCSTR_20440, and KsCSTR_20420 (kuste3556), which encode the proteins of MoxR-like ATPase in aerotolerance operon, ABC-type uncharacterized transport system, and putative ABC-type transport system (ATPase component), respectively, were also downregulated (*p*-adj < 0.05). Conversely, only the genes KsCSTR_25000 encoding Clp ATPase C-terminal domain-containing protein and *exeA* (KsCSTR_21550, KsCSTR_31550, KsCSTR_35740, and KsCSTR_25860) were upregulated (*p*-adj < 0.05) (Table 2 and Fig. 3b). The *exeA* describes the AAA (ATPases Associated with diverse cellular Activities) protein family, which is responsible for providing energy to catalyze ATP hydrolysis (Miller and Enemark 2016). During the N_2_H_4_ exposure, anammox bacteria may need to utilize a larger energy supply in order to maintain their integrity. Additionally, the stress response gene (*hsp*) was significantly downregulated as a result of exogenous N_2_H_4_ addition (*p*-adj < 0.01) (Table 2). The decrease in expression of *hsp* may be a result of decreased ATP production when energy metabolism is suppressed (Yan et al. 2020). Furthermore, *HtrA* proteases play a key role in bacterial stress responses, preventing cellular damage and restoring cellular homeostasis by refolding or degrading misfolded proteins that accumulate due to heat, oxidative stress, or acidic conditions (Xue et al. 2021). In this study, the upregulation of KsCSTR_24920 (*p*-adj < 0.05) likely reflects a stress response to elevated N_2_H_4_ levels.

##### *CO*_*2*_* fixation, nitrite reduction and substrate trafficking*

Anammox bacteria fix atmospheric CO_2_ through the acetyl-CoA pathway. It has been demonstrated that the enzyme acetyl-CoA synthase (*acs*ABCD) is employed in the genome of *K. stuttgartiensis* (Strous et al. 2006). Two genes encoding the beta and delta subunits (*acsB*, KsCSTR_09660 and *acsD*, KsCSTR_09730) were downregulated by N_2_H_4_ exposure (*p*-adj < 0.05). Moreover, the expression levels of other important genes involved in nitrogen metabolism, including KsCSTR_08000 (*narG*), KsCSTR_14610 (*focA*), and KsCSTR_41990 (*nirD/nirG*) are also decreased by N_2_H_4_ addition to the anammox systems (*p*-adj < 0.01) (Table 2).

The largest operon involved in fatty acid biosynthesis in *K. stuttgartiensis* is the kuste3352-kuste3335 gene cluster (Strous et al. 2006). Within this cluster, kuste3335 (KsCSTR_17470) catalyzes the final step in ladderane glycerophospholipid synthesis by esterifying the ladderane fatty acyl group to L-glycerol 3-phosphate (Rattray et al. 2009). In this study, kuste3335 was significantly upregulated under N_2_H_4_ exposure (*p*-adj < 0.05). Other gene clusters implicated in fatty acid biosynthesis include kuste3606-3608, kuste1390-1386, and kuste2802-2805, some of which encode type II fatty acid biosynthesis enzymes such as *fabF*, *fabG*, *fabH*, *fabZ*, etc. (Rattray et al. 2009). Among them, kuste3605 (KsCSTR_20950), located in a highly expressed native cluster (Javidpour et al. 2016), was downregulated (*p*-adj < 0.05) in this study. Conversely, kuste3346 (KsCSTR_17580), part of the largest gene cluster, was one of the most upregulated genes in anammox metabolism (Fig. 3b). In a previous study, upregulation of *fabF* transcription has been linked to fatty acid synthesis inhibition in *Bacillus subtilis* (Schujman Gustavo et al. 2001). Similarly, the marked upregulation of fatty acid biosynthesis genes in this study suggests that N_2_H_4_ exposure may have inhibited anammox bacterial metabolism.

Additionally, NO is another critical intermediate in the anammox process, commonly produced by reducing nitrite to NO (Eq. 1) (Kartal and Keltjens 2016). This reaction is catalyzed by one of two enzymes: the heme protein NirS (*cd*_1_ nitrite reductase) or the copper-containing NirK. In *K. stuttgartiensis*, the genome contains structural genes for heme *c* and *d*_1_. However, these genes are rarely expressed at significant levels at the transcriptional and protein levels (Kartal and Keltjens 2016); thus, they are barely detectable. In the current study, no changes were observed in the expression of genes coding for these enzymes. Anammox bacteria may also utilize different enzymes capable of producing NO. HAO-like kustc0458, KsCSTR_29630, and KsCSTR_43280 could be possible candidates (Kartal et al. 2013). In this study, as mentioned before in 3.2.3.1, two of them (KsCSTR_29630 and KsCSTR_43280) were downregulated by a high level of N_2_H_4_ exposure (Table 2). On the other hand, the nitrite removal rate also increased as the applied N_2_H_4_ dosage was elevated (Fig. 1b). Thus, anammox bacteria may have developed multiple pathways for generating NO, considering NO is an essential intermediate in metabolism. It may have provided them with metabolic flexibility to adapt to changing environmental conditions (Kartal et al. 2013). This versatility may be crucial for their survival and efficiency in different operational scenarios.

Although N_2_H_4_ is typically found in low concentrations in environmental samples, its concentration can reach up to 200 mg/L, particularly in layup solutions used to protect idle boilers (ASTM 2018). N_2_H_4_ is toxic and may exhibit carcinogenic properties at certain concentrations (NTP 2021), making its removal from wastewater a critical concern. In addition to the current applications of anammox bacteria, these bacteria can tolerate elevated N_2_H_4_ concentrations (up to 1.88 g/L) during short-term exposure (“Short-Term Response to Hydrazine” section in the current study). Therefore, anammox bacteria can be practically utilized for N_2_H_4_ removal from industrial wastewaters. Our previous study has demonstrated that anammox bacteria have great potential for treating N_2_H_4_-containing wastewater, alongside their widespread environmental applications (Sari et al. 2024b). On day 162 during the reactor operation (L2) (a total of 3.71 g/L of N_2_H_4_ cumulative load) (Sari et al. 2024b), MLVSS was also collected from the bioreactor for metatranscriptome analysis. The results uncovered that there was no significant change in the gene expression profile related to the central anammox metabolism (unpublished data), indicating the adaptation of the enriched anammox culture to long-term N_2_H_4_ exposure. However, overall findings of the current study have shown that anammox bacteria are susceptible to shock loads of N_2_H_4_ at high concentrations. Numerous genes involved in central anammox metabolism and energy metabolism, as well as anammox anabolism, were downregulated at a statistically significant level (Table 2). Hence, a drastic increase in the effluent N_2_H_4_ concentration can cause a deterioration in anammox activity.

The current study also emphasizes the importance of monitoring and controlling N_2_H_4_ concentrations to prevent its detrimental effects on anammox bacteria. Industrial effluents often contain N_2_H_4_ concentrations that may exceed the threshold tolerated by anammox bacteria, leading to a loss of their nitrogen removal efficiency. Consequently, this study provides valuable insights for optimizing wastewater treatment processes that utilize anammox bacteria, especially in industries where N_2_H_4_-containing wastewater is prevalent. Future research should focus on identifying the specific N_2_H_4_ concentration threshold that leads to significant inhibition of anammox bacteria and on developing strategies to enhance their tolerance to N_2_H_4_. These strategies may include genetic modifications, optimization of operational parameters, or the development of microbial consortia capable of tolerating and degrading high N_2_H_4_ concentrations. Ultimately, such strategies would improve the robustness and efficiency of anammox-based systems, enabling effective nitrogen removal even in environments containing inhibitory compounds like hydrazine.

Literature studies have also suggested that anammox is a promising technology for N_2_H_4_ biosynthesis (Oshiki et al. 2020; Sari et al. 2024a). However, anammox metabolism is constrained at high N_2_H_4_ concentrations, which likely limits both the efficiency of N_2_H_4_ production and the survival of anammox bacteria. Therefore, for sustainable N_2_H_4_ bioproduction, it is crucial to investigate the response of anammox bacteria to relatively lower N_2_H_4_ concentrations. Furthermore, a deeper understanding of anammox metabolism is essential for enhancing its potential in both N_2_H_4_ removal and biosynthesis. However, genetic manipulation of anammox bacteria remains a significant challenge due to their slow growth rate, low biomass yield, and sensitivity to environmental conditions. Pure cultures of anammox bacteria have not yet been obtained, limiting the ability to study their metabolic pathways and optimize their practical applications (Peeters and van Niftrik 2019; Pereira et al. 2017). Overcoming these challenges will be essential for advancing anammox-based biotechnological processes in future research.

## Conclusions

In this study, the short-term responses of the enriched anammox culture to exogenous N_2_H_4_ at concentrations higher than previously reported in the literature were investigated. The nitrogen removal profiles revealed that anammox bacteria can tolerate N_2_H_4_ concentrations up to approximately 1.88 g/L and even metabolize N_2_H_4_. Notably, exogenous N_2_H_4_ enhanced NO_2_^−^-N consumption efficiency and rate of the anammox bacteria. However, NH_4_^+^-N started to accumulate in the system, potentially due to N_2_H_4_ disproportionation reactions in metabolism. Furthermore, acute exposure to high N_2_H_4_ concentrations significantly downregulated key genes involved in central anammox and energy metabolisms, such as *hzs*, *hdh*, *hao*, and so on, except for AAA family ATPase. This indicates that anammox bacteria may require an increased energy supply to maintain cellular integrity under N_2_H_4_ stress. Although the anammox process in this study is considered a new approach beyond its conventional uses, the findings demonstrate that excessive N₂H₄ concentrations may severely impair anammox activity at a molecular level. Future studies should focus on strategies to enhance anammox adaptability to high N_2_H_4_ levels, which could expand their applicability in engineered wastewater treatment and biotechnological processes.

## Supplementary Information

Below is the link to the electronic supplementary material.

Supplementary file1 (DOCX 1565 KB)

Supplementary file2 (XLSX 49 KB)

Supplementary file3 (XLSX 16 KB)

## Data Availability

All data supporting the findings of this study are available within the paper and its Supplementary Information.
