# Correction: Physiological and transcriptomic response of enriched anammox culture upon elevated hydrazine exposure

**DOI:** 10.1007/s10532-025-10151-3

**Published:** 2025-07-08

**Authors:** Tugba Sari, Kozet Yapsakli, Deniz Akgul, Bulent Mertoglu

**Affiliations:** 1 Department of Bioengineering, Marmara University, Goztepe, 34722 Istanbul, Türkiye; 2 Department of Environmental Engineering, Marmara University, Goztepe, 34722 Istanbul, Türkiye

**Correction: Biodegradation** 10.1007/s10532-025-10132-6

In Table 2 of this article, the header “Gene DESCRIPTION” should be written as in sentence case: “Gene description”. Additionally, the header line should be boldfaced should be as below.

**Table 2:**
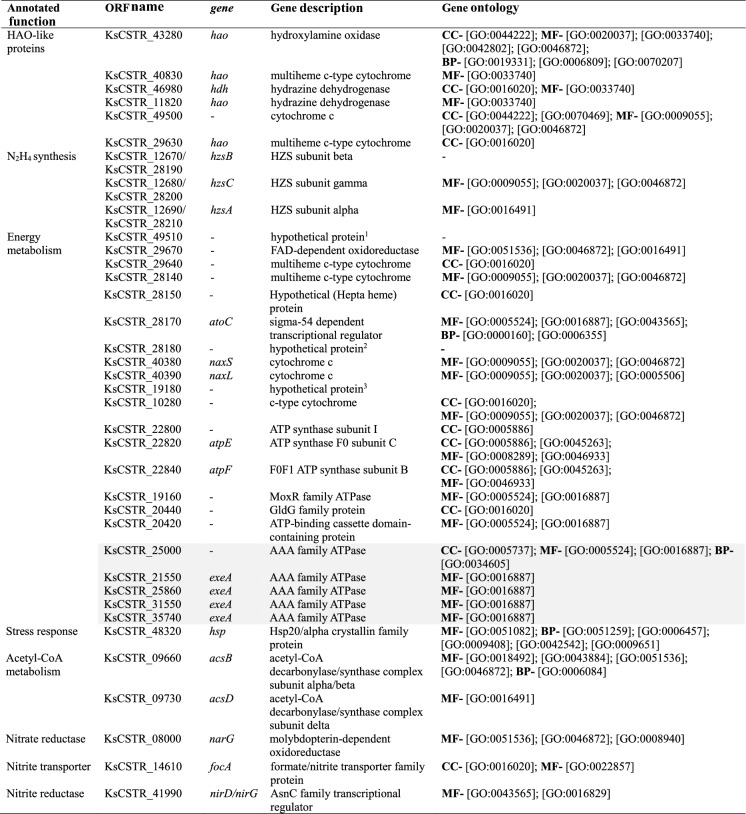
Overview of the most important anammox genes detected in the transcriptomes

Highlighted genes were upregulated, others were downregulated. Gene names and gene ontology were obtained from UniProt database. CC: Cellular component; MF: Molecular function; BP: Biological process. ^1^submitted protein name: Type-1 blue copper-containing cupredoxin; ^2^ submitted protein name: Uncharacterized protein; ^3^ submitted protein name: C-type heme protein

The original article has been corrected.

