# Supplementary material for: Physiological and transcriptomic response of enriched anammox culture upon elevated hydrazine exposure
Source: Biodegradation. 2025 May 5;36(3):39. doi: 10.1007/s10532-025-10132-6 (PMC12053348; doi:10.1007/s10532-025-10132-6)
Supplement: Supplementary file 1 — Supplementary file1 (DOCX 1565 KB) [file 10532_2025_10132_MOESM1_ESM.docx]

**Supplementary Information-1**

**Table S1.** Basic alignment statistics for sequencing reads mapped to the *Candidatus* Kuenenia stuttgartiensis reference genome (ASM1106654v1, GCF_011066545.1) per sample.

| **Sampe Name** | **Mapped Read Number** | **Genome Mapping Ratio (%)** |
| --- | --- | --- |
| CONTROL1.bam | 5521940 | 71.11 |
| CONTROL2.bam | 2702364 | 81.99 |
| TREAT1.bam | 6780333 | 89.10 |
| TREAT2.bam | 7717523 | 75.49 |

**Table S2.** Software and Tools used in bioinformatics analysis.

| **Softwares/Tools** | **Version** | **Function** | **Ref.** |
| --- | --- | --- | --- |
| FastQC | v0.12.1 | “.fastq” Quality Evaluation and Visualization | (Brown et al. 2017) |
| MultiQC | v1.19.0 | “.fastq” Quality Evaluation and Visualization | (Ewels et al. 2016) |
| fastp | v0.23.4 | Raw “.fastq” Sequence Preprocessing | (Chen et al. 2018) |
| trim_galore | v0.6.10 | Raw “.fastq” Sequence Preprocessing | (Krueger 2015) |
| Samtools | v1.19.0 | The alignment file (“.sam” and “.bam”) manipulation and metric collections | (Li et al. 2009) |
| Sambamba | v0.8.2 | The alignment file (“.sam” and “.bam”) manipulation and metric collections | (Tarasov et al. 2015) |
| FeatureCounts | v2.0.7 | Gene/Transcript abundance estimation | (Liao et al. 2014) |
| DeSeq2 | v1.42.0 | Differential expression analysis | (Love et al. 2014) |
| Blast | v2.15.0 | Sequence similarity search | (Ye et al. 2006) |
| Hmmer | v3.4.0 | Sequence similarity search | (Mistry et al. 2013) |
| ClustVis | v1.01 | Data Visualization | (Metsalu and Vilo 2015) |
| topGO | v2.54.0 | Functional Enrichment (GO & Pathway) | (Alexa and Rahnenführer 2009) |
| g:Profiler | v0.2.2 | Functional Enrichment (GO & Pathway) | (Kolberg et al. 2020) |
| EnrichmentMap | v3.3.5 | Functional Enrichment (GO & Pathway) | (Reimand et al. 2019) |
| Kraken2 | v2.1.3 | Taxonomic Classification | (Wood et al. 2019) |
| Silva Database | 138.1 update  release | A comprehensive on-line resource for ribosomal RNA sequence data. | (Quast et al. 2012) |

**Text S1.** GO enrichment analysis for differentially expressed genes upon N_2_H_4_ exposure.

GO is the most well-known database and functional classification system for genes, which provides a set of annotations to comprehensively describe the properties of genes and gene products. GO is divided into three main categories: biological process (BP), molecular function (MF), and cellular component (CC). As a result of the analysis, a total of 75 biological processes, 17 cellular components, and 24 molecular functions were identified. The genes identified in biological processes are mostly involved in cellular and/or metabolic processes, metabolic and biosynthetic processes related to (cellular) nitrogen compounds and organic substances, and metabolic and biosynthetic processes related to (cellular) macromolecules. They are followed by gene expression, translation, protein and/or peptide metabolic processes, and amide metabolic and biosynthetic processes. As categories of molecular function, the genes were detected to be mainly responsible for binding, organic cyclic compound binding, heterocyclic compound binding, followed by nucleic acid binding, RNA binding, structural molecule activity, and structural constituents of the ribosome. In addition, the cellular anatomical entity was found to be a category of cellular component in which genes are mostly involved. For the whole list, see SI 3.

**
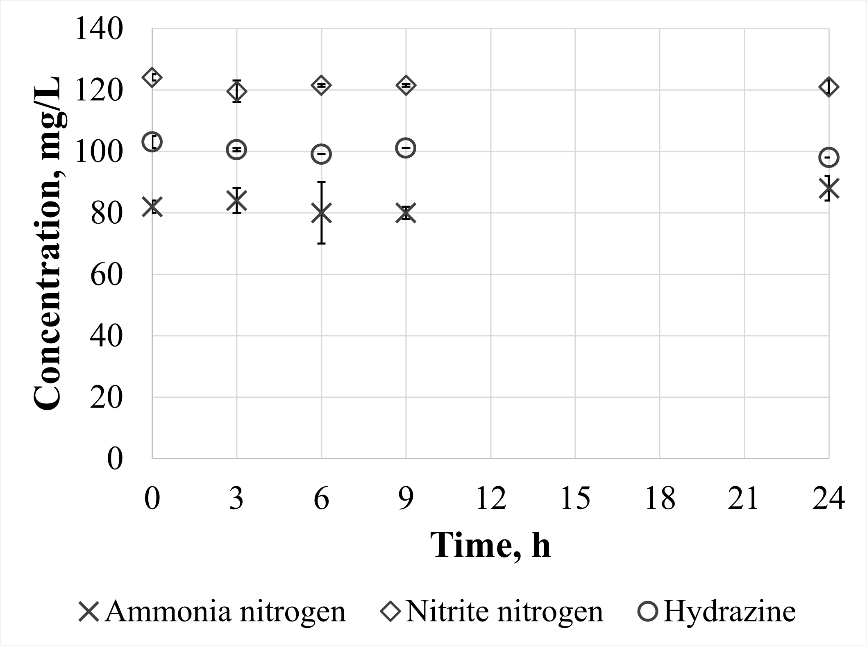
**

**Fig. S1.** Negative control experiment.

1.
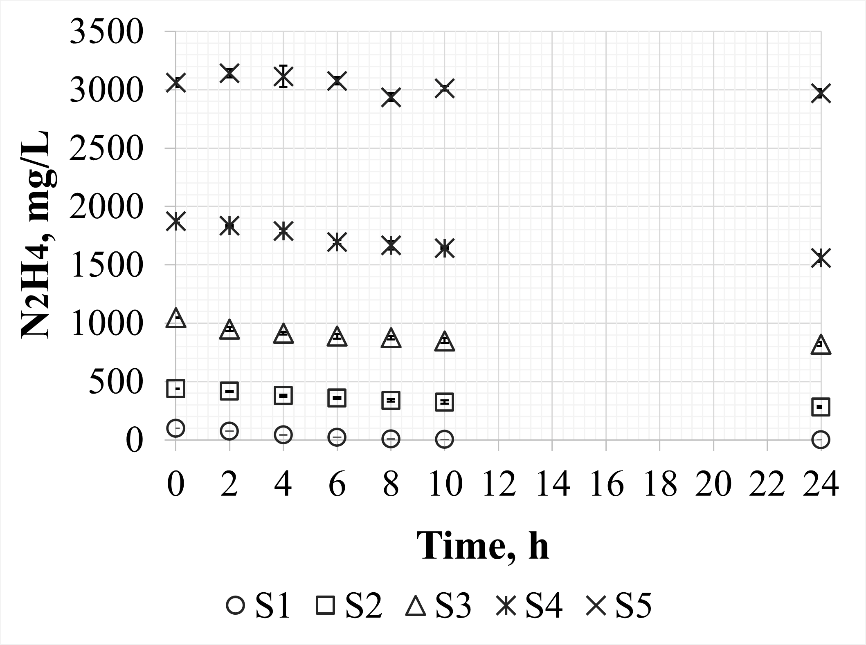

2.
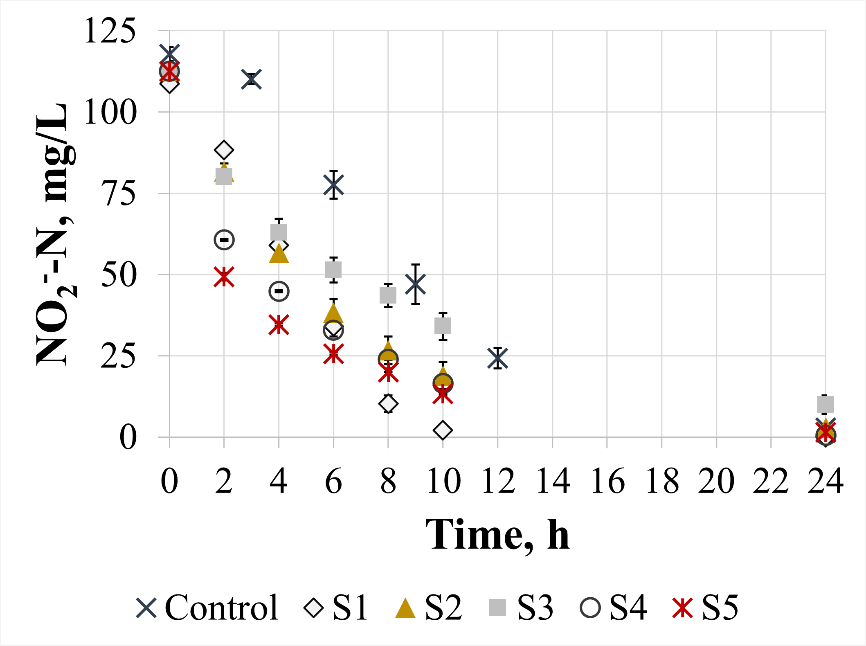

3.
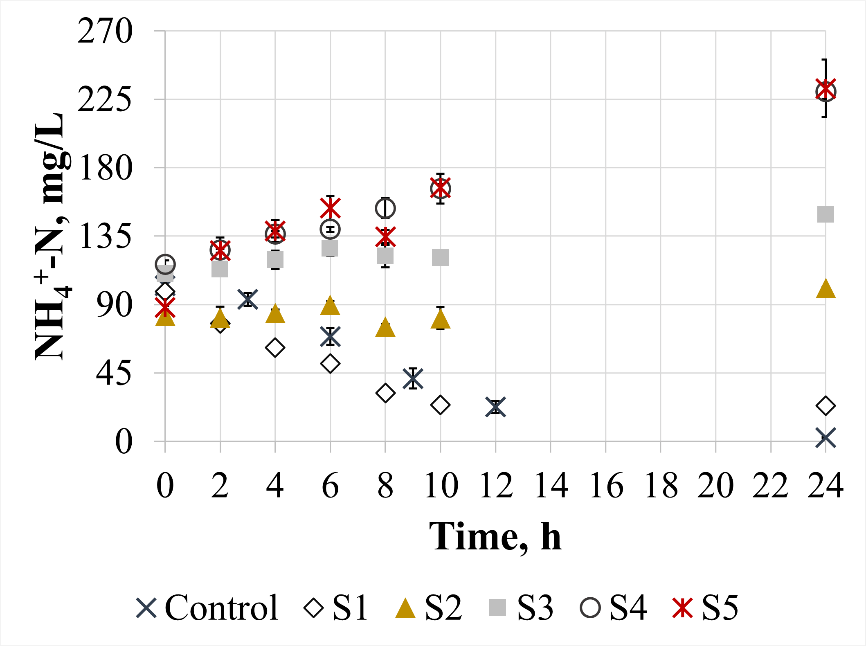


**Fig. S2.** Nitrogenous components consumption profiles in batch tests. a) hydrazine; b) nitrite nitrogen; c) ammonia nitrogen

1. **
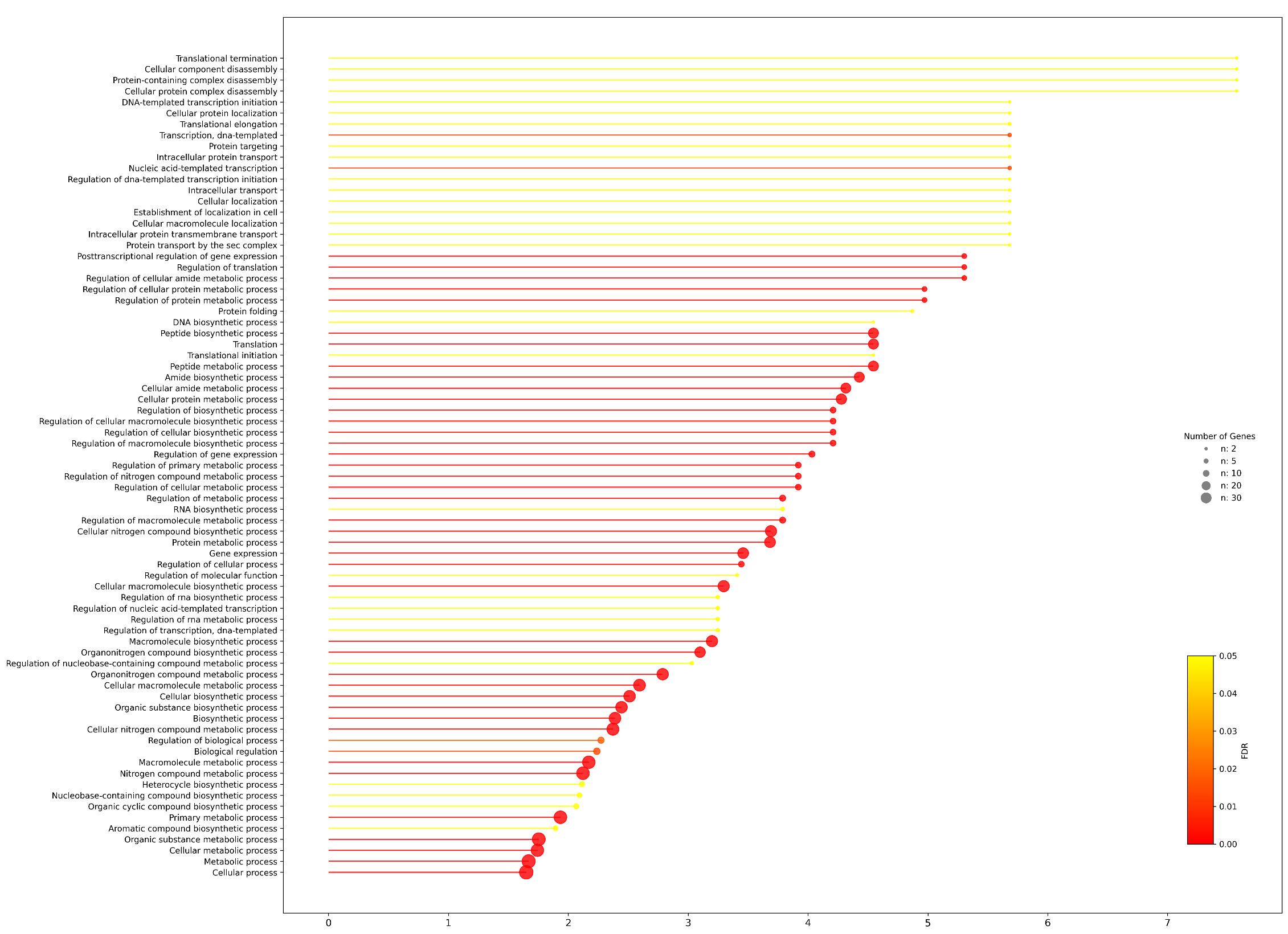
**
2. **
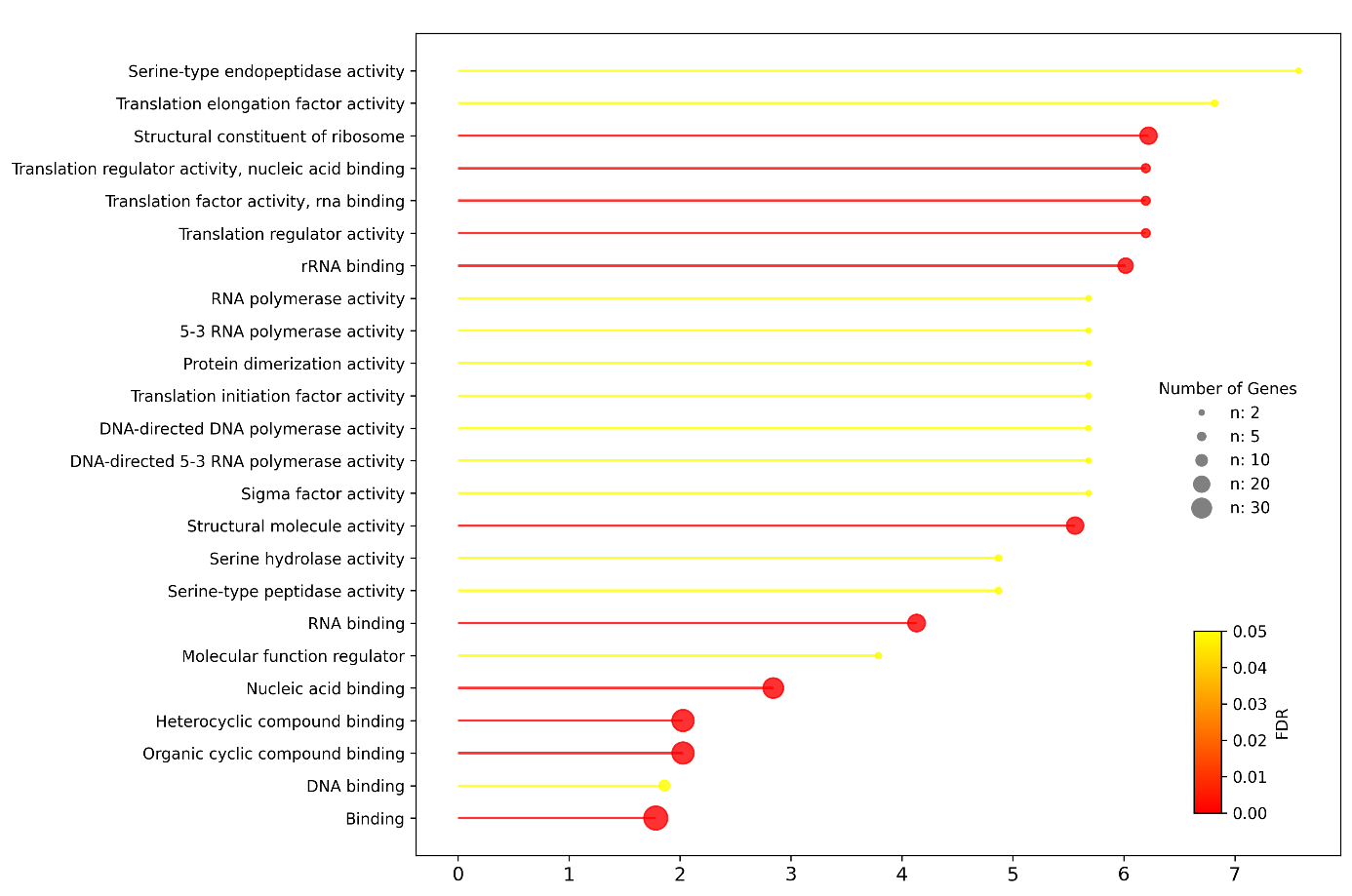
**
3. **
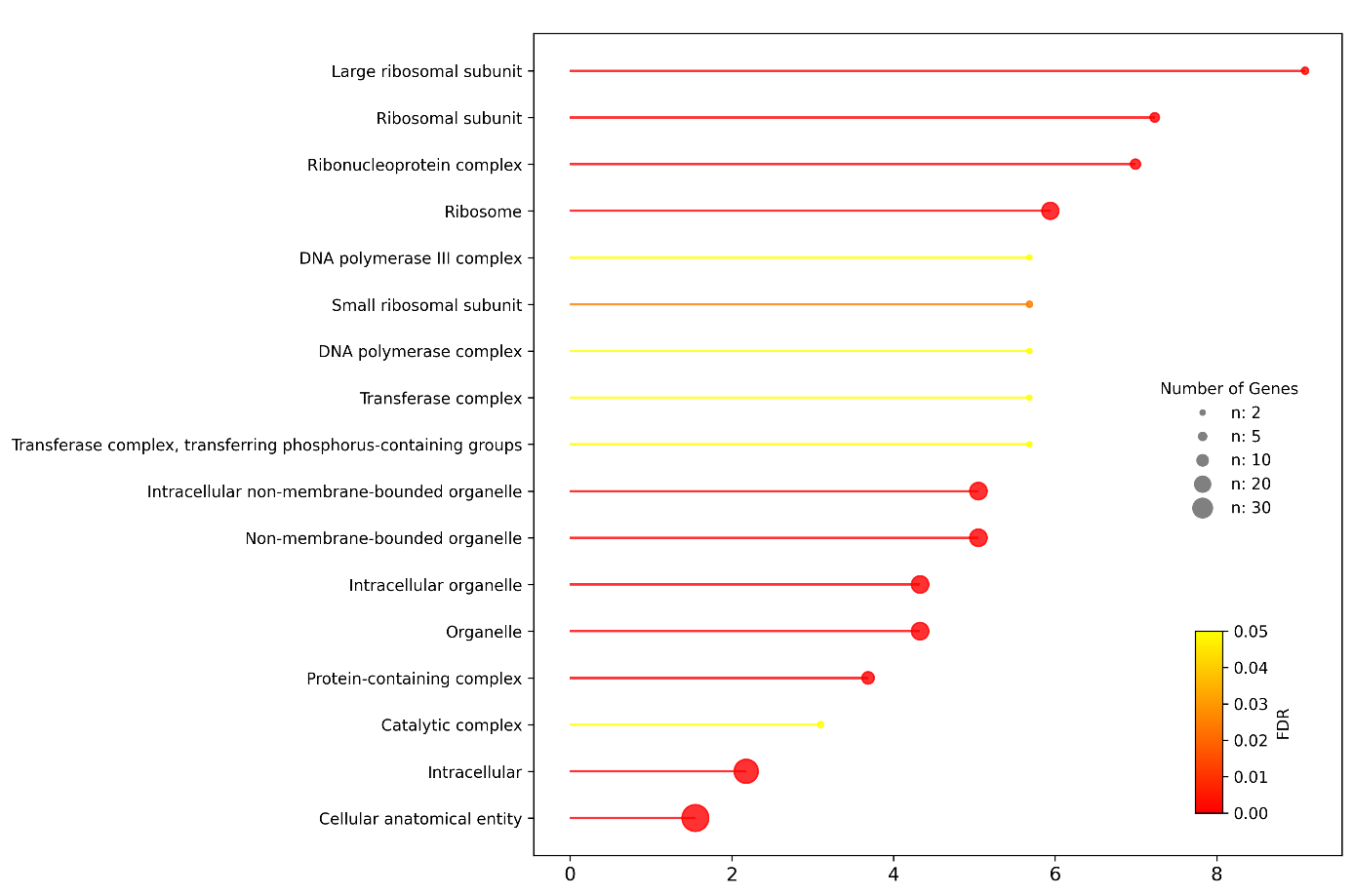
**

**Fig. S3.** Analysis of GO enrichment for DEGs. a) biological processes, b) molecular function, c) cellular component. FDR stands for the false discovery rate, which measures the proportion of false discoveries among a set of hypothesis tests called significant.

**References**

Alexa A, Rahnenführer J (2009) Gene set enrichment analysis with topGO. Bioconductor Improv 27:1-26.

Brown J, Pirrung M, McCue LA (2017) FQC Dashboard: integrates FastQC results into a web-based, interactive, and extensible FASTQ quality control tool. Bioinformatics 33:3137-3139. <https://doi.org/10.1093/bioinformatics/btx373>

Chen S, Zhou Y, Chen Y, Gu J (2018) fastp: an ultra-fast all-in-one FASTQ preprocessor. Bioinformatics 34:i884-i890. <https://doi.org/10.1093/bioinformatics/bty560>

Ewels P, Magnusson M, Lundin S, Käller M (2016) MultiQC: summarize analysis results for multiple tools and samples in a single report. Bioinformatics 32:3047-3048. <https://doi.org/10.1093/bioinformatics/btw354>

Kolberg L, Raudvere U, Kuzmin I, Vilo J, Peterson H (2020) gprofiler2--an R package for gene list functional enrichment analysis and namespace conversion toolset g: Profiler. F1000Research 9.

Krueger F (2015) Trim Galore!: A wrapper around Cutadapt and FastQC to consistently apply adapter and quality trimming to FastQ files, with extra functionality for RRBS data. Babraham Institute.

Li H, Handsaker B, Wysoker A, Fennell T, Ruan J, Homer N, Marth G, Abecasis G, Durbin R (2009) The Sequence Alignment/Map format and SAMtools. Bioinformatics 25:2078-2079. <https://doi.org/10.1093/bioinformatics/btp352>

Liao Y, Smyth GK, Shi W (2014) featureCounts: an efficient general purpose program for assigning sequence reads to genomic features. Bioinformatics 30:923-930. <https://doi.org/10.1093/bioinformatics/btt656>

Love M, Anders S, Huber W (2014) Differential analysis of count data–the DESeq2 package. Genome Biol. 15:10-1186.

Metsalu T, Vilo J (2015) ClustVis: a web tool for visualizing clustering of multivariate data using Principal Component Analysis and heatmap. Nucleic Acids Res. 43:W566-W570.

Mistry J, Finn RD, Eddy SR, Bateman A, Punta M (2013) Challenges in homology search: HMMER3 and convergent evolution of coiled-coil regions. Nucleic Acids Res. 41:e121-e121.

Quast C, Pruesse E, Yilmaz P, Gerken J, Schweer T, Yarza P, Peplies J, Glöckner FO (2012) The SILVA ribosomal RNA gene database project: improved data processing and web-based tools. Nucleic Acids Res. 41:D590-D596.

Reimand J, Isserlin R, Voisin V, Kucera M, Tannus-Lopes C, Rostamianfar A, Wadi L, Meyer M, Wong J, Xu C (2019) Pathway enrichment analysis and visualization of omics data using g: Profiler, GSEA, Cytoscape and EnrichmentMap. Nat. Protoc. 14:482-517.

Tarasov A, Vilella AJ, Cuppen E, Nijman IJ, Prins P (2015) Sambamba: fast processing of NGS alignment formats. Bioinformatics 31:2032-2034.

Wood DE, Lu J, Langmead B (2019) Improved metagenomic analysis with Kraken 2. Genome Biol. 20:257. <https://doi.org/10.1186/s13059-019-1891-0>

Ye J, McGinnis S, Madden TL (2006) BLAST: improvements for better sequence analysis. Nucleic Acids Res. 34:W6-W9.
